# Drug-Resistant Polymorphisms and Copy Numbers in *Plasmodium falciparum*, Mozambique, 2015

**DOI:** 10.3201/eid2401.170864

**Published:** 2018-01

**Authors:** Himanshu Gupta, Eusebio Macete, Helder Bulo, Crizolgo Salvador, Marian Warsame, Eva Carvalho, Didier Ménard, Pascal Ringwald, Quique Bassat, Sonia Enosse, Alfredo Mayor

**Affiliations:** ISGlobal, Barcelona Institute for Global Health Hospital Clínic, Universitat de Barcelona, Barcelona, Spain (H. Gupta, Q. Bassat, A. Mayor);; Centro de Investigação em Saúde de Manhiça, Maputo, Mozambique (E. Macete, H. Bulo, Q. Bassat, A. Mayor);; Instituto Nacional de Saúde, Ministério da Saúde, Maputo (C. Salvador, S. Enosse);; World Health Organization Global Malaria Programme, Geneva, Switzerland (M. Warsame, P. Ringwald);; World Health Organization, Maputo (E. Carvalho);; Malaria Molecular 16 Epidemiology Unit, Institut Pasteur du Cambodge, Phnom Penh, Cambodia (D. Ménard);; Institut Pasteur, Paris, France (D. Ménard); ICREA, Barcelona (Q. Bassat);; Pediatrics Department, Hospital Sant Joan de Déu (University of Barcelona);; Barcelona, Spain (Q. Bassat)

**Keywords:** antimicrobial resistance, *K13*, plasmepsin2, polymorphisms, copy number, Mozambique, malaria, parasites, vector-borne infections

## Abstract

One of the fundamental steps toward malaria control is the use of antimalarial drugs. The success of antimalarial treatment can be affected by the presence of drug-resistant populations of *Plasmodium falciparum*. To assess resistance, we used molecular methods to examine 351 *P. falciparum* isolates collected from 4 sentinel sites in Mozambique for *K13*, *pfmdr1*, *pfcrt*, and *pfdhps* polymorphisms and for *plasmepsin2* (*pfpm2*) and *pfmdr1* copy numbers. We found multiple copies of *pfpm2* in 1.1% of isolates. All isolates carried *K13* wild-type alleles (3D7-like), except 4 novel polymorphisms (Leu619Leu, Phe656Ile, Val666Val, Gly690Gly). Prevalence of isolates with *pfcrt* mutant (K76T) allele was low (2.3%). Prevalence of isolates with *pfdhps* mutant alleles (A437G and K540E) was >80%, indicating persistence of sulfadoxine/pyrimethamine resistance; however, markers of artemisinin were absent, and markers of piperaquine resistance were low. Piperaquine resistance isolates may spread in Mozambique as dihydroartemisinin/piperaquine drug pressure increases.

During the past decade, malaria control strategies have substantially reduced the malaria burden worldwide; several countries are advancing toward malaria elimination (*1*,*2*). A fundamental pillar for contributing to the reduction of the malaria burden has been artemisinin-based combination therapy. Unfortunately, the effectiveness of antimalarial drugs used for malaria treatment and chemoprevention during pregnancy has been threatened by the emergence of drug-resistant parasite populations (*2*–*5*).

The emergence of artemisinin resistance in *Plasmodium falciparum*, with reduced in vivo susceptibility to artesunate, was reported in Southeast Asia (*3*,*6*). Detectable polymorphisms in the *Kelch 13* (*K13*) propeller domain in *P. falciparum* associated with artemisinin resistance have subsequently provided an additional tool for monitoring resistance to antimalarial drugs (*7*,*8*). In Cambodia, polymorphisms in the *K13* propeller domain (mainly Y493H, R539T, I543T, and C580Y) were associated with in vitro prolonged parasite survival rates and in vivo delayed parasite clearance rates (*8*,*9*). Recently, *plasmepsin 2* (*pfpm2*) copy number and *pfcrt* C101F polymorphism have been associated with piperaquine resistance (*10*–*12*). In addition, increased *pfmdr1* copies have been associated with resistance to mefloquine (in vivo, in vitro, or both) and partially to lumefantrine (*13*–*18*). Specific point polymorphisms (at codons 86, 184, 1034, 1042, and 1246) of the *pfmdr1* gene have also been linked to resistance to antimalarial drugs (*19*,*20*). In field isolates tested in vitro as well as in laboratory lines, N86Y polymorphism was associated with chloroquine resistance (*21*). Further, polymorphisms in the *pfcrt* gene have also been shown to affect parasite susceptibility to chloroquine (*22*), amodiaquine (*23*,*24*), and artemether/lumefantrine (*25*). Recently, a nonsynonymous polymorphism in the *pfcrt* gene was shown to be prevalent in the genetic background of *K13* mutant artemisinin-resistant isolates (*26*). In addition, polymorphisms in *pfdhfr* and *pfdhps* genes, specifically the quintuple mutant, including the *pfdhfr* substitutions N51I, C59R, and S108N, as well as the *pfdhps* substitutions A437G and K540E, have been associated with a failure of sulfadoxine/pyrimethamine treatment against uncomplicated *P. falciparum* malaria (*27*). In Africa, the *pfdhps* K540E polymorphism has been considered a useful epidemiologic marker of the quintuple mutations (*28*).

The development of drug resistance could be influenced by multiple factors such as polymorphism rate, fitness costs, overall parasite load, strength of drug selection, treatment compliance, transmission intensity, host immunity, and erythrocyte disorders (*29*–*31*). Naturally acquired immunity plays a major role in the emergence and clearance of artemisinin-resistant parasites (*32*). Because of increasing concern over the effectiveness of the nationally recommended antimalarial drugs, the Mozambique Ministry of Health has made several changes in antimalarial drug policy. In 2002, chloroquine monotherapy was replaced with sulfadoxine/pyrimethamine/amodiaquine as the first line of treatment against uncomplicated malaria (*33*); 2 years later, this combination was replaced with artesunate/sulfadoxine/pyrimethamine (*33*). In 2008, artemether/lumefantrine was introduced to replace artesunate/sulfadoxine/pyrimethamine (*34*). Molecular markers for antimalarial drug resistance have been considered useful for confirming parasite resistance, a major factor causing treatment failure. To determine whether parasites carrying these polymorphisms or gene amplifications exist in Mozambique, we conducted molecular surveillance targeting *K13*, *pfmdr1*, *pfcrt*, and *pfdhps* polymorphisms and *pfpm2* and *pfmdr1* copy numbers in field isolates collected from 4 sentinel sites.

## Materials and Methods

### Study Sites and Population

**Figure 1:**
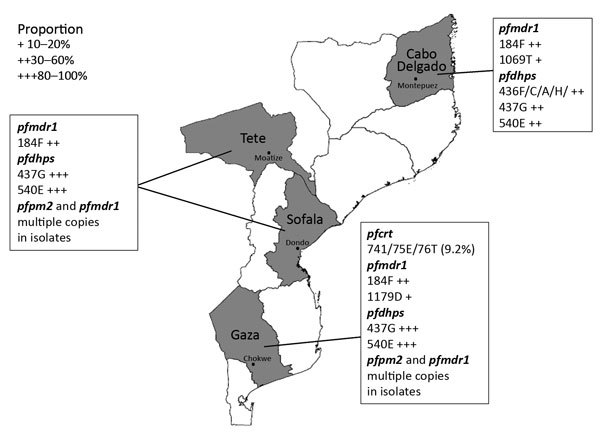
Location of sampling sites and distribution of resistance markers of *Plasmodium falciparum* in Mozambique, 2015.

We performed a descriptive observational study on blood samples collected before artemether/lumefantrine treatment (on day 0) in 2015 from 352 symptomatic children at 4 sentinel sites in Mozambique (Figure 1): 1) Hospital Rural de Montepuez in Cabo Delgado Province (northern region), 2) Centro de Saúde de Dondo in Sofala Province (central region), 3) Hospital Provincial de Moatize in Tete Province (central region), and 4) Hospital Rural de Chokwe in Gaza Province (southern region). In Mozambique, transmission usually peaks during the rainy season (November–April). Transmission intensity in southern Mozambique is generally low, although areas of high transmission may still occur (*35*). To determine molecular markers of drug resistance, we analyzed samples collected during a clinical trial conducted in 2015 (registration no. ACTRN12616001680459); the trial aimed to assess the efficacy and safety of artemether/lumefantrine for treatment of uncomplicated *P. falciparum* malaria in children <5 years of age. The National Mozambican Ethical Review Committee (Mozambique) and Hospital Clínic (Barcelona, Spain) ethics review committees approved the study, and signed written informed consent was obtained from all participants’ guardian or parent.

### Molecular Procedures

We extracted DNA from half of a 50-μL dried blood drop on Whatman 3-mm filter paper by using a QIAamp DNA Mini kit (QIAGEN, Hilden, Germany) according to the manufacturer’s instructions. We used an ABI PRISM 7500 HT Real-Time System (Applied Biosystems, Foster City, CA, USA) to amplify purified parasite DNA templates, following a previously described method (*36*,*37*). A standard curve was prepared from an in vitro culture of 3D7 strain containing known numbers of ring-infected erythrocytes. The standard curve was run in triplicate for each test with 5 serially diluted points. Parasitemia in the clinical samples was quantified by extrapolation against the standard curve.

To assess polymorphisms in the *K13, pfcrt*, *pfmdr1*, and *pfdhps* genes, we amplified purified DNA templates by using a 2720 Thermal Cycler (Applied Biosystems), following protocols described for *K13* PCR (*38*) and *pfcrt* PCR (*35*). To genotype polymorphisms in *pfmdr1* and *pfdhps* genes, we designed new assays by using Sanger sequencing and restriction fragment length polymorphisms (Technical Appendix). A total of 6 positive controls with known *K13* alleles, provided by the Institut Pasteur in Cambodia, and 4 parasite lines (3D7, 7G8, Dd2, and V1/S) with known *pfcrt* and *pfmdr1* alleles, available in the laboratory, were also processed, amplified, and sequenced at the same time as the studied samples (PCR characteristics in Technical Appendix Table). To determine the detection limit of Sanger sequencing, we used artificially mixed DNA samples of *P. falciparum* laboratory strains containing various known proportions of wild- and mutant-type alleles of *pfcrt* (K76T) and *pfmdr1* (Y184F and S1034C) genes. To estimate polymorphism frequency, we considered isolates with mixed alleles to be mutated.

We assessed copy numbers of *pfpm2* and *pfmdr1* genes as described elsewhere (*11*) with minor changes (Technical Appendix) by using quantitative PCR (qPCR). We performed amplification in 20-μL reaction mixtures for *pfpm2*, *pfmdr1*, and *pfβ-tubulin* genes, separately. We used the *pfβ*-tubulin gene as an endogenous control. All samples with estimated copy numbers >1.5 were defined as containing multiple copies and repeated for confirmation. The estimated copy numbers were the average of the copy number of each clone in the isolate.

### Data Analyses

We calculated the proportion of the mutant alleles and isolates with multiple copies of *pfpm2* and *pfmdr1* genes on the basis of the number of samples with wild- and mutant-type alleles as well as isolates with single and multiple copies of the gene from *P. falciparum* isolates from each study site. To compare continuous data and categorical data between sites, respectively, we performed analyses of variance and χ^2^ tests. We defined statistical significance as p<0.05.

## Results

### Demographics and *P. falciparum* Infection

Among the 352 blood samples collected before artemether/lumefantrine treatment (on day 0) during 2015, and followed up as part of the clinical trial, 351 (99.7%) were *P. falciparum–*infection positive according to 18SrRNA qPCR. The mean (± SD) parasitemia (by qPCR) was 100,229 ± 325,214 parasites/μL. Among participants, 159 (45.2%) were female, mean (± SD) age was 2.8 ± 1.3 y, mean body temperature was 38.1 ± 1.1°C, and mean hemoglobin level was 9.2 ± 1.9 g/dL. We also compared demographic data and parasite densities according to study site (Table 1). Efficacy of artemether/lumefantrine in the in vivo study was high, and for nearly all patients (349 [99.4%] of 351), parasitemia reverted to 0 in the first 3 days; however, for 2 patients, parasites were still detectable by microscopy: 1 from Moatize (514 parasites/μL) and 1 from Chokwe (3,763 parasites/μL). PCRs targeting *msp1*, *msp2*, and *glurp* genes were used to differentiate recrudescence (same parasite strain) and reinfection (different parasite strain). We noted recrudescence of *P. falciparum* infections for 5 children (1 in Chokwe, 3 in Moatize, and 1 in Montepuez) on days 21 and 28 after artemether/lumefantrine administration and reinfection for 7 children (3 in Moatize and 4 in Montepuez); 3 were reinfected on day 21 and 4 on day 28 (*39*).

**Table 1:** Characteristics of study participants with *Plasmodium falciparum* malaria, by site, Mozambique, 2015

Characteristic	Montepuez, n = 87	Dondo, n = 88	Moatize, n = 89	Chokwe, n = 88	p value
Female, no. (%)	36 (41.4)	40 (45.5)	41 (46.1)	42 (47.7)	0.63
Age, y, mean ± SD	2.4 ± 1.1	2.7 ± 1.1	2.7 ± 1.1	3.1 ± 1.1	0.0002
Temperature, °C, mean ± SD	37.9 ± 0.9	38.5 ± 1.1	38.1 ± 1.0	37.9 ± 1.5	0.0043
Parasite density, parasites/μL, mean ± SD*	1.2 × 10^5^ ± 1.3 × 10^5^	3.7 × 10^4^ ± 4.4 × 10^4^	1.2 × 10^5^ ± 6.1 × 10^5^	1.2 × 10^5^ ± 1.8 × 10^5^	<0.0001
Hemoglobin, g/dL, mean ± SD	8.9 ± 2.2	8.7 ± 1.8	9.8 ± 1.7	9.2 ± 2.0	0.0018

*By quantitative PCR.

The polymorphism analyses of *K13*, *pfmdr1, pfcrt*, and *pfdhps* genes were successful for 98.3% to 100% isolates. Because no amplifications were noticed in negative controls (with water and human genomic DNA), PCR assays were specific to *P. falciparum* genomic DNA only.

### Detection Limit of Mixed Samples by Sanger Sequencing

We identified ¨A¨ alleles of *pfcrt* (K76T) and *pfmdr1* (Y184F) codons in artificially mixed samples by using Sanger sequencing when the proportion of target DNA was >10%. However, we identified ¨C¨ and ¨T¨ alleles of *pfcrt* (K76T) and *pfmdr1* (Y184F) polymorphisms, respectively, in mixed samples when their proportion was >20% (Technical Appendix Figure 1). For *pfmdr1* (S1034C) polymorphism, the minor allele was detected when its proportion was >20% in a mixed sample (Technical Appendix Figure 1, panel C). For positive controls, we used several parasite lines with known *K13*, *pfmdr1*, and *pfcrt* alleles. As expected, sequencing analysis of all positive controls revealed wild- and mutant-type alleles of *K13*, *pfmdr1*, and *pfcrt* polymorphisms.

### Copy Numbers for *pfpm2* and *pfmdr1*

**Figure 2:**
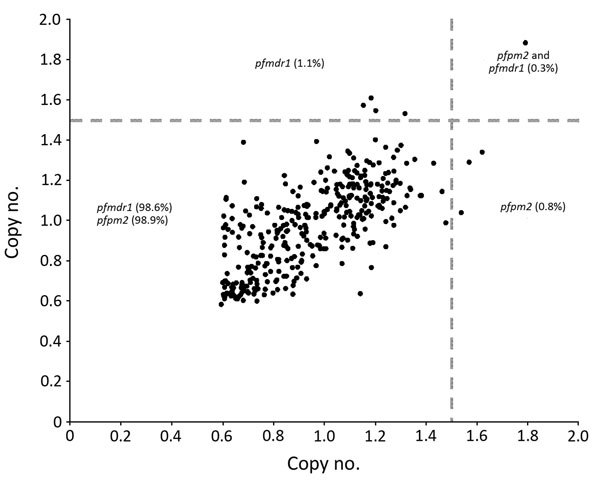
*pfpm2* and *pfmdr1* copy numbers of *Plasmodium falciparum* isolates from 4 sentinel sites, Mozambique, 2015. Multiple copies of *pfpm2* and *pfmdr1* genes have been associated with resistance to piperaquine and mefloquine, respectively.

We successfully analyzed 351 (100%) samples for copy number variation in the *pfpm2* and *pfmdr1* genes. PCR efficiencies were 98.4% for *pfpm2*, 97.2% for *pfmdr1*, and 99.2% for *pfβ-tubulin* genes. As expected, the estimated *pfpm2* and *pfmdr1* copy numbers for the positive controls were 3–4 copies. The estimated mean (interquartile range) copy numbers were 3.51 (3.37–3.62) for *pfpm2* and 3.62 (3.51–3.79) for *pfmdr1* positive controls. When we used a copy number threshold of 1.5 to define multiple copies, only 4 (1.1%) and 5 (1.4%) of the 351 isolates had multiple copies of *pfpm2* and *pfmdr1*, respectively (Table 2; Figure 2). The range of estimated *pfpm2* copy numbers was 0.59–1.79 and of *pfmdr1* was 0.58–1.88. The copy number of *pfpm2* and *pfmdr1* genes did not significantly differ between isolates from different sites. The proportion of isolates with multiple copies of the *pfpm2* gene was the highest at Chokwe (2 [2.3%] of 87). Only 1 (1.1%) of 88 samples from Dondo had multiple copies of *pfpm2* and *pfmdr1* genes.

**Table 2:** *Plasmodium falciparum* isolates with increased *pfpm2* and *pfmdr1* gene copy numbers, 4 sentinel sites, Mozambique, 2015

Site	*pfpm2*					*pfmdr1*			
No. (%)			Mean ± SD	No. (%)			Mean ± SD
<1.2	1.2–1.5	>1.5		<1.2	1.2–1.5	>1.5	
Montepuez, n = 87	82 (94.3)	5 (5.7)	0	0.85 ± 0.2		87 (100.0)	0	0	0.93 ± 0.1
Dondo, n = 88	66 (75.0)	21 (23.9)	1 (1.1)	1.02 ± 0.2		68 (77.3)	18 (20.4)	2 (2.3)	0.99 ± 0.3
Moatize, n = 89	73 (82.0)	15 (16.9)	1 (1.1)	1.01 ± 0.2		81 (91.0)	7 (7.9)	1 (1.1)	0.99 ± 0.2
Chokwe, n = 87	77 (88.5)	8 (9.2)	2 (2.3)	0.94 ± 0.2		70 (80.5)	15 (17.2)	2 (2.3)	0.98 ± 0.2

### *K13* Polymorphisms

We successfully achieved *K13* PCR and sequencing for all 351 isolates. None of the isolates analyzed contained the polymorphisms most frequently found in isolates from Cambodia (*8*). However, we observed 4 novel polymorphisms at nt 1725147 (codon 619; 0.28% [1/351]); 1725032 (codon 656; 0.28% [1/351]); 1725000 (codon 666; 0.57% [2/351]); and 1724927 (codon 690; 0.85% [3/351]) of the *K13* gene. All polymorphisms were synonymous except for 1 at codon 656, which led to a change from phenylalanine to isoleucine. When we compared frequencies of new polymorphisms between sites, we found no significant differences. We also observed the polymorphism Cys469Cys, previously described in *P. falciparum* field isolates from Ghana (*40*), in 3 (0.85%) of the 351 isolates. Isolates from patients with parasitemia on day 3 and recrudescence contained wild-type *K13* gene polymorphisms.

### *pfcrt* Polymorphisms

We successfully amplified all 351 samples for *pfcrt* and sequenced the amplification products; mutant alleles were found at codons M74I, N75E, and K76T only in 8 (2.3%) samples. The mutant alleles (M74I, N75E, and K76T) were present only in isolates collected from Chokwe (8 [9.2%] of 87). When we compared frequencies of mutant alleles between sites, the difference was significant (p<0.0001). In the studied isolates, the mutant (F) allele at codon 101 was absent. Isolates from patients with parasitemia on day 3 and recrudescence contained wild-type *pfcrt* gene polymorphisms.

### *pfmdr1* Polymorphisms

We successfully amplified and sequenced *pfmdr1*_f1 for 351 (100%) samples and *pfmdr1*_f2 fragments for 350 (99.7%) samples. We identified 15 polymorphisms all across the *pfmdr1* gene, including 5 (33.3%) with nonsynonymous polymorphisms and 10 (66.7%) with synonymous polymorphisms. Among nonsynonymous polymorphisms, 3 (T1192A, F1194S, and Y1197N) were newly identified and 2 (N86Y and Y184F) had been previously reported (*41*). Among synonymous polymorphisms, 7 (L1030L, D1061D, D1127D, S1137S, L1174L, D1179D, and N1189N) were newly identified and 3 (G102G, G182G, and T1069T) had been previously reported (*41*). Among the 351 isolates, we found 11 (3.1%) N86Y and 164 (46.7%) Y184F mutant alleles (Table 3). All newly identified nonsynonymous mutant alleles were present only once, except for Y1197N, which was found twice (0.6% [2/350]). The frequency of polymorphisms (N86Y and D1179D) differed significantly between isolates from the 4 sites (Table 3). The proportion of N86Y and D1179D polymorphisms was highest in isolates from Chokwe. We observed none of the other most frequent polymorphisms (S1034C, N1042D, and D1246Y) of the *pfmdr1* gene among the analyzed samples. Isolates from patients with parasitemia on day 3 and recrudescence contained wild-type *pfmdr1* gene polymorphisms.

**Table 3:** Distribution of *Plasmodium falciparum* *pfmdr1* polymorphism (mutated allele) frequencies among 4 sentinel sites, Mozambique, 2015*

SNP	Montepuez, no. (%)	Chokwe, no. (%)	Moatize, no. (%)	Dondo, no. (%)	p value
N86Y	1 (1.1)	6 (6.9)	0	4 (4.5)	0.03
G102G	1 (1.1)	2 (2.3)	2 (2.2)	1 (1.1)	0.88
G182G	4 (4.6)	1 (1.1)	3 (3.4)	1 (1.1)	0.37
Y184F	45 (51.7)	39 (44.8)	42 (47.2)	38 (43.2)	0.69
L1030L	0	0	1 (1.1)	0	0.40
D1061D	0	1 (1.1)	0	0	0.40
T1069T	10 (11.5)	5 (5.7)	4 (4.5)	8 (9.2)	0.28
D1127D	1 (1.1)	0	2 (2.2)	0	0.30
S1137S	2 (2.3)	0	0	2 (2.3)	0.25
L1174L	1 (1.1)	0	0	0	0.40
D1179D	0	9 (10.3)	2 (2.2)	0	0.0001
N1189N	0	0	1 (1.1)	1 (1.1)	0.57
T1192A	0	1 (1.1)	0	0	0.40
F1194S	1 (1.1)	0	0	0	0.40
Y1197N	1 (1.1)	0	0	1 (1.1)	0.57

*SNP, single-nucleotide polymorphism.

### *pfdhps* Polymorphisms

Polymorphism analysis by PCR followed by sequencing for S436F and A437G polymorphisms was successful for 345 (98.3%) samples and analysis by PCR–restricted fragment length polymorphism for K540E polymorphism for 348 (99.1%) samples. Among all isolates, 10 (2.9%) of 345 contained S436F, 289 (83.8%) of 345 contained A437G, and 286 (82.2%) of 348 contained K540E mutant alleles. At codon 436, we also found 3 mutant alleles: S436C (0.9%), S436A (4.9%), and S436H (0.6%). When we compared frequencies of 3 single-nucleotide polymorphisms at different sites, we noted significant differences (Table 4). The proportion of isolates with A437G and K540E polymorphisms was the highest at Moatize, and the proportion with S436F, S436C, S436A, and S436H alleles was highest at Montepuez.

**Table 4:** Distribution of *Plasmodium falciparum* *pfdhps* gene polymorphism (mutated allele) frequencies among 4 sentinel sites, Mozambique, 2015*

SNP	Montepuez, no. (%)	Chokwe, no. (%)	Moatize, no. (%)	Dondo, no. (%)	p value
S436F/C/A/H	30 (34.5)	2 (2.4)	0	0	<0.0001
A437G	51 (58.6)	79 (94.1)	82 (93.2)	77 (89.5)	<0.0001
K540E	50 (57.5)	77 (90.6)	83 (93.3)	76 (87.4)	<0.0001
A437G + K540E	47 (54.1)	77 (91.7)	80 (90.9)	76 (88.4)	<0.0001
*SNP, single-nucleotide polymorphism.					

## Discussion

We provide evidence for the presence of multiple copies of *pfpm2* in 4 (1.1%) of 351 *P. falciparum* isolates circulating in southern Mozambique despite the absence of piperaquine drug pressure. Thus, with adequate drug pressure, isolates resistant to piperaquine may spread in Mozambique, as occurred in Southeast Asia (*10*,*42*,*43*). In selected areas of Cambodia in 2008, piperaquine was introduced as a partner drug of artemisinin (*44*). Soon after its introduction, as early as 2010, piperaquine resistance in western Cambodia emerged at an alarming rate (*45*). Subsequent reports confirmed a rapid increase in failure of dihydroartemisinin/piperaquine in other parts of Cambodia (*42*,*46*,*47*). The most frequent *K13* mutants associated with artemisinin resistance were absent in the isolates from Mozambique. We also determined that prevalence of *pfcrt* (K76T) and *pfmdr1* (N86Y) markers of resistance are low, supporting previous evidence for the return of parasites carrying *pfcrt* wild-type alleles in Mozambique (*35*), in contrast to persistence of *pfdhps* (A437G [83.8%]) and K540E [82.2%]) polymorphisms, markers of sulfadoxine/pyrimethamine resistance (*34*). The well-characterized polymorphism in *pfmdr1* (Y184F [46.7%]) was also prevalent in Mozambique.

We found very low prevalence (<1%) for 4 new polymorphisms (Leu619Leu, Phe656Ile, Val666Val, and Gly690Gly) in the *K13* gene of *P. falciparum* isolates from Mozambique. All polymorphisms except Phe656Ile were synonymous. Previously, V494I *K13* nonsynonymous polymorphism has also been reported in Mozambique (*48*). In Africa, *K13* nonsynonymous polymorphisms have also been reported at low frequencies in isolates from Cameroon, Central African Republic, Democratic Republic of the Congo, Gabon, The Gambia, Kenya, Madagascar, Malawi, Mali, Rwanda, Togo, Uganda, Zambia, and Equatorial Guinea (*38*,*40*,*49*–*50*; references 51,52 in Technical Appendix). The association of nonsynonymous polymorphisms with delayed parasite clearance has only recently been identified in Africa (reference 52 in Technical Appendix).

Resistance to both chloroquine and amodiaquine has been mainly associated with a single K76T mutant allele in the *pfcrt* gene (*22*–*24*). In our study, its prevalence in 8 (2.3%) of 351 samples was significantly lower than that found in previous studies in Mozambique (*33*,*34*; reference 53 in online Technical Appendix). Our *pfcrt* data align with previous evidence for the return of parasites carrying *pfcrt* wild-type alleles in Mozambique (*35*) and in other countries in Africa, such as Ethiopia (reference 54 in Technical Appendix), Malawi (reference 55 in Technical Appendix), and Cameroon (reference 56 in Technical Appendix). The selective disadvantage of mutant parasites in the absence of drug pressure has been proposed as the leading factor contributing to the reemergence of chloroquine-susceptible parasites (reference 57 in Technical Appendix). Because artemether/lumefantrine has been shown to select for the wild-type *pfcrt* 76K allele (*25*), this reemergence might be accelerated because of the increased use of artemether/lumefantrine as a first-line treatment for uncomplicated malaria in Mozambique (reference 53 in Technical Appendix). 

Our study also provides evidence for the presence of few *P. falciparum* isolates with multiple copies of the *pfmdr1* gene (5 [1.4%] of 351) circulating in southern Mozambique (*34*). Increased *pfmdr1* copies have been associated with resistance to mefloquine and partial resistance to lumefantrine (*13*–*18*). Our study found that prevalence of the *pfmdr1* N86Y mutant allele has decreased and the Y184F mutant allele has increased over time, in contrast with findings of other studies from Mozambique (*34*; references 55,58 in Technical Appendix). We identified 10 new polymorphisms (L1030L, D1061D, D1127D, S1137S, L1174L, D1179D, N1189N, T1192A, F1194S, and Y1197N) that had not been previously described for the *pfmdr1* gene. Among the 15 polymorphisms identified in the *pfmdr1* gene, we observed significant differences between sites for the N86Y and D1179D polymorphisms only.

Of 351 children who had received adequate treatment with artemether/lumefantrine (6 doses), 2 were still positive for parasitemia on day 3 (*39*). These isolates contained wild-type *K13* gene polymorphisms. *P. falciparum–*positive patients for whom artemether/lumefantrine treatment failed had parasites that carried wild-type *pfcrt* and *pfmdr1* polymorphisms. This observation suggests that in vivo artemether/lumefantrine resistance may be caused not only by variations in the *pfcrt* and *pfmdr1* genes but possibly by parasite selection of variations in other genes; however, drug bioavailability issues may also have contributed.

A high proportion of the *P. falciparum* isolates from Mozambique contained K540E (82.2%) and A437G (83.8%) mutant alleles. These mutant alleles may still not jeopardize the effectiveness of sulfadoxine/pyrimethamine for malaria prevention in Mozambique; recent findings suggest that only >90% prevalence of a *pfdhps* K540E polymorphism could reduce the effectiveness of intermittent preventive therapy to clear peripheral parasites and prevent new infections during pregnancy (reference 59 in Technical Appendix). Therefore, sulfadoxine/pyrimethamine remains effective for intermittent preventive therapy during pregnancy, despite the high frequency of quintuple mutants; thus, the World Health Organization continues to recommend the use of intermittent preventive therapy to prevent malaria during pregnancy (references 60–62 in Technical Appendix). However, alternative antimalarial drugs for intermittent preventive therapy during pregnancy are needed because the prevalence of the K540E polymorphism in Mozambique is close to the threshold.

In conclusion, we report that prevalence of isolates with multiple copies of *pfpm2* is lower than that found by previous studies in Cambodia (34.3%) and Vietnam (54.3%) (*10*,*43*), and we report the absence of *K13* polymorphisms known to be associated with artemisinin resistance. We also report the return of parasites carrying *pfcrt* wild-type alleles (except in Chokwe) and persistence of parasites with *pfdhps* mutations associated with sulfadoxine/pyrimethamine resistance in Mozambique. Sulfadoxine/pyrimethamine–resistant isolates may be maintained by the constant use of intermittent preventive therapy during pregnancy, use of drug outside of hospitals, the very common use of co-trimoxazole (as prophylaxis for HIV-infected persons), and the low fitness cost of the polymorphisms (*33*; references 63,64 in Technical Appendix). In contrast, the fitness cost of the *pfcrt* mutant allele seems to be high, probably accounting for the return of parasites carrying *pfcrt* wild-type alleles in Mozambique (reference 57 in Technical Appendix). Current regional elimination efforts, as part of the G8 Malaria Elimination Initiative, may lead to more aggressive strategies involving population-wide distribution of antimalarial drugs, such as dihydroartemisinin/piperaquine, resulting in significantly increased drug pressure. Our findings might provide baseline prevalence data that enable us to directly determine the effects that increasing malaria control efforts or elimination programs will have on resistance evolution.

Technical Appendix Sanger sequencing details, sequence of the oligonucleotides used for PCR and sequencing, assessment of the detection limit by Sanger sequencing in artificially mixed DNA samples containing various proportions of *pfcrt* (K76T) and *pfmdr1* (Y184F and S1034C) alleles, and additional text references.
