# Supplementary material for: Drug-Resistant Polymorphisms and Copy Numbers in Plasmodium falciparum, Mozambique, 2015
Source: Emerg Infect Dis. 2018 Jan;24(1):40–8. doi: 10.3201/eid2401.170864 (PMC5749434; doi:10.3201/eid2401.170864)
Supplement: Technical Appendix — Sanger sequencing details, sequence of the oligonucleotides used for PCR and sequencing, assessment of the detection limit by Sanger sequencing in artificially mixed DNA samples containing various proportions of pfcrt (K76T) and pfmdr1 (Y184F and S1034C) alleles, and additional text references. [file 17-0864-Techapp-s1.pdf]

Article DOI: http://dx.doi.org/10.3201/eid2401.170864

Drug-Resistant Polymorphisms and Copy Numbers in *Plasmodium falciparum*, Mozambique, 2015

Technical Appendix

Sanger Sequencing

pfmdr1 polymorphisms

pfmdr1 gene was divided into two separate fragments (*pfmdr1*_f1 and *pfmdr1*_f2). The *pfmdr*_f1 and *pfmdr*_f2 fragments were amplified separately in 25µl reactions including 5µl of template DNA, 0.5µM of each forward and reverse specific primer set for *pfmdr*_f1 and *pfmdr*_f2 fragments (Technical Appendix Table) and 1xHOT FirePol Master Mix (Solis BioDyne; Riia, Tartu, Estonia), reaction volume was raised by PCR-grade water. In a thermocycler, both the fragment amplification followed the same PCR conditions, the template DNA was denatured at 95°C for 15min, followed by 35 cycles of amplification (95°C for 1min, 60°C for 1min, and 72°C for 1min 30s) and a final extension at 72°C for 10min. PCR products were run on 2% agarose (Invitrogen, Carlsbad, CA, USA) gels in 1× TBE buffer (Invitrogen, Carlsbad, CA, USA) to determine the presence and size of the amplified DNA and PCR products were visualized using a UV trans-illuminator. The expected size of PCR products for *pfmdr1*_f1 and *pfmdr1*_f2 fragments was 610bp and 1295bp respectively. Whenever enough amplified products were not obtained, re-PCR were performed using the same primer sets and PCR conditions mentioned above for both *pfmdr1*_f1 and *pfmdr1*_f2 fragments, 5µl of PCR products were used as DNA templates for re-PCRs. To sequence the f1 and f2 fragments of *pfmdr1* gene, two new pairs of sequencing primers were used (Technical Appendix Table). The sequencing primer pair, specific for *pfmdr1*_f1 fragment, covers 45 – 209 aa and *pfmdr1*_f2 specific sequencing primer pair covered 984 – 1277 aa of PfMDR1 protein. The specificity of all primer sets was tested against human genomic DNA and no DNA template control.

pfdhps **polymorphisms**

For *pfdhps* gene amplification, a single PCR based assay was designed. In brief, amplification was performed in 25µl reaction including 5µl of template DNA, 0.5µM of each forward and reverse specific primer set for *pfdhps* (Technical Appendix Table) and 1x HOT FirePol Master Mix, reaction volume was raised by PCR-grade water. The template DNA was denatured at 95°C for 15min in a thermocycler, followed by 32 cycles of amplification (95°C for 1min, 54°C for 1min, and 72°C for 1min) and a final extension at 72°C for 10 min. The expected size of the PCR product was 875bp was visualized as mentioned above covering 357- 647 aa of PfDHPS protein. PCR products were sequenced using a forward primer of PCR and new reverse sequencing primer (Technical Appendix Table) covering 357 – 477 aa of PfDHPS protein. Positive and negative (water instead of template DNA) controls were added in every PCRs and plates. The specificity of all primer sets was tested against human genomic DNA and no DNA template control.

For bi-directional sequencing, ≈1200ng of PCR products (quantified using EPOCH Biotech system) were sent to Genewiz (Takeley, United Kingdom), following safety instructions for the accurate shipment of PCR amplicons. The variations in the test sequences of *K13, pfcrt, pfmdr1* and *pfdhps* were identified by sequence alignment against PF3D7_1343700, PF3D7_0709000, PF3D7_0523000 and PF3D7_0810800 reference sequence of 3D7 respectively, retrieved from PlasmoDB.

pfdhps **- Restriction fragment length polymorphism**

To detect the K540E polymorphism in *pfdhps,* the restriction fragment length polymorphism (RFLP) was performed. The PCR products of 875bp (described above) of *Pfdhps* gene were digested with FokI enzyme (New England Biolabs, Ipswich, MA, USA) as per manufacture instruction's which generated 538bp, 315bp and 22bp fragments whenever mutant allele was present, and two fragments of 853bp and 22bp were seen for wild type allele.

pfmdr1 **and** ***pfpm2*** **gene copy number - qPCR**

The quantitative PCR (qRT-PCR) was used to assess variations in the copy number (CN) of *pfpm2* and *pfmdr1* genes as described elsewhere (*1*) with minor changes. In brief, 96-well plates were prepared containing separate 20µl reaction mixtures for *pfpm2, pfmdr1* and *pfβ-tubulin* genes. *Pfβ-tubulin* gene was used as an endogenous control. Each reaction mixture had

2X Power SYBR® Green PCR Master Mix (Thermo Fisher Scientific, Warrington, WA, UK), 0.25 μM of each forward and reverse primers and 5μl of template DNA, reaction volume was makeup by PCR-grade water. Amplifications were performed with a holding for 3mins at 50°C, initial denaturation for 10mins at 95°C, followed by 40 cycles of 95°C for 15s and 58°C for 45s, extra dissociation stage was added to ensure the specificity of primer pairs. The 20μl reaction mixtures in a 96-well plate were amplified in a 7500 HT Real-Time System (Applied Biosystem, Foster City, USA). For each run, the *pfpm2* and *pfmdr1* copy numbers of each sample were measured in triplicate. The PCR efficiencies of the *pfpm2*, *pfmdr1* and *pfβ-tubulin* genes were measured using ten-fold dilutions of 3D7 DNA. The specificity of three primer pairs against human genomic DNA was also determined. Along with no template control, we also included one positive control with the known 3-4 copies of *pfpm2* gene provided by Dr. Didier Ménard (Pasteur Institute, Cambodia) and another positive control for *pfmdr1* gene, genomic DNA of DD2 parasite line with known 3-4 copies of *pfmdr1*. All samples with C_t >33 for *pfpm2*, *pfmdr1* and *pfβ-tubulin* were not considered for the CN analysis. The *pfmdr1* and *pfpm2* genes CNs were estimated as described previously (*1*). All samples with estimated CN values above 1.5 were confirmed two more times.

Reference

1. Witkowski B, Duru V, Khim N, Ross LS, Saintpierre B, Beghain J et al. A surrogate marker of piperaquine-resistant *Plasmodium falciparum* malaria: a phenotype-genotype association study. Lancet Infect Dis. 2017;17:174–83. http://dx.doi.org/10.1016/S1473-3099(16)30415-7

Technical Appendix Table. The sequence of the oligonucleotides used for PCR and sequencing, PCR annealing temperature (Tm), several cycles, the size of amplicons and covered amino acids

	Conventional PCR				Sequencing	
Gene	PCR primers	Annealing Temp (°C)	Cycles	PCR size (bp)	Sequencing primers	Covered amino acids
K13	OuterF:cggagtgaccaaatctggga	58	30	2097	F:gccaagctgccattcatttg	427–709
	OuterR:gggaatctggtggtaacagc					
	NestedF:gccaagctgccattcatttg	60	40	849	R:gccttgttgaaagaagcaga	
	NestedR:gccttgttgaaagaagcaga					
pfcrt	OuterF:tttaggtggaggttcttgtctt	52	25	569	F:tttaggtggaggttcttgtctt	35–120
	OuterR:atacttaattgaagaacaaatgattgga					
	NestedF:tttaggtggaggttcttgtctt	50	35	319	R:ttggtaggtggaatagattctct	
	NestedR:ttggtaggtggaatagattctct					
*pfmdr1*_F1	F:agagaaaaaagatggtaacctcag	60	35	610	F:ccgtttaaatgtttacctgcac	45–209
	R:accacaaacataaattaacgg				R:accacaaacataaattaacgg	
*pfmdr1*_F2	F:accccaggtgttttatctgc	60	35	1295	F:tttttaatacaggaagcatttt	984–1277
	R:tgtccacctgataagcttttacc				R:aatgttgcatcttctcttc	
pfdhps	F:tgtgagtaggatgaaagaacaa	54	32	875	F:tgtgagtaggatgaaagaacaa	357–477
	R:tccaattgtgtgatttgtcca				R:attggtttcgcatcacattt	

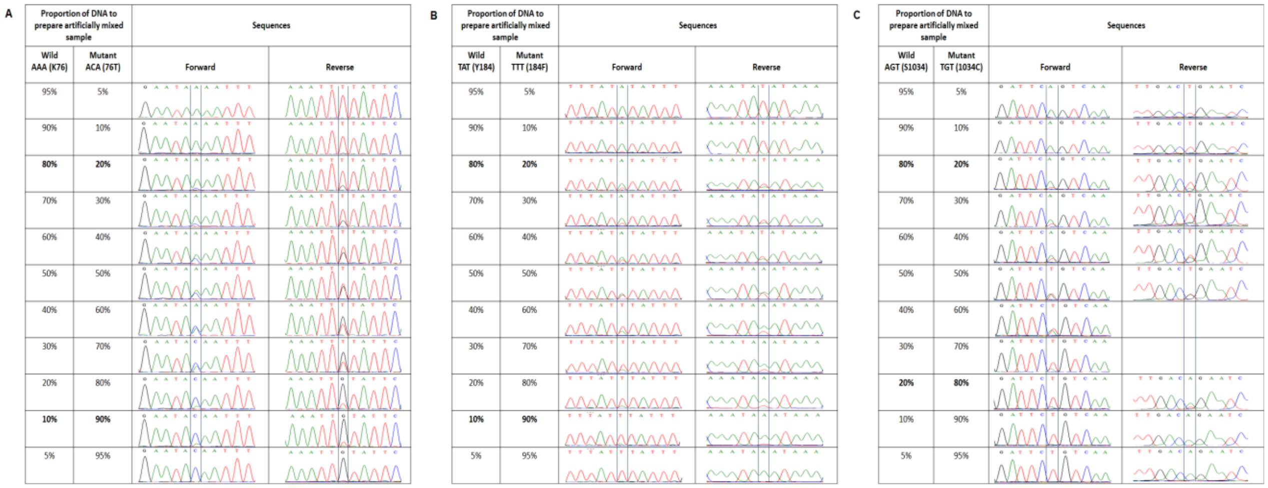

Technical Appendix Figure. Assessment of the detection limit by Sanger sequencing in artificially mixed DNA samples, containing various proportions of *pfcrt* (K76T) and *pfmdr1* (Y184F and S1034C) alleles. Panel A: K76T polymorphism of *pfcrt* gene; Panel B and C is for Y184F and S1034C polymorphisms of *pfmdr1* gene, respectively.

Additional Text References

51. Torrentino-Madamet M, Fall B, Benoit N, Camara C, Amalvict R, Fall M, et al. Limited polymorphisms in k13 gene in Plasmodium falciparum isolates from Dakar, Senegal in 2012-2013. Malar J. 2014;13:472. PubMed http://dx.doi.org/10.1186/1475-2875-13-472

52. Lu F, Culleton R, Zhang M, Ramaprasad A, von Seidlein L, Zhou H et al. Emergence of indigenous artemisinin-resistant *Plasmodium falciparum* in Africa. N Engl J Med. 2017; 376:991–53. http://dx.doi.org/10.1056/NEJMc1612765

53. Thomsen TT, Madsen LB, Hansson HH, Tomás EV, Charlwood D, Bygbjerg IC, et al. Rapid selection of Plasmodium falciparum chloroquine resistance transporter gene and multidrug resistance gene-1 haplotypes associated with past chloroquine and present artemether-lumefantrine use in Inhambane District, southern Mozambique. Am J Trop Med Hyg. 2013;88:536–41. PubMed http://dx.doi.org/10.4269/ajtmh.12-0525

54. Mekonnen SK, Aseffa A, Berhe N, Teklehaymanot T, Clouse RM, Gebru T, et al. Return of chloroquine-sensitive Plasmodium falciparum parasites and emergence of chloroquine-resistant Plasmodium vivax in Ethiopia. Malar J. 2014;13:244. PubMed http://dx.doi.org/10.1186/1475-2875-13-244

55. Frosch AE, Laufer MK, Mathanga DP, Takala-Harrison S, Skarbinski J, Claassen CW, et al. Return of widespread chloroquine-sensitive Plasmodium falciparum to Malawi. J Infect Dis. 2014;210:1110–4. PubMed http://dx.doi.org/10.1093/infdis/jiu216

56. Ndam NT, Basco LK, Ngane VF, Ayouba A, Ngolle EM, Deloron P, et al. Reemergence of chloroquine-sensitive pfcrt K76 Plasmodium falciparum genotype in southeastern Cameroon. Malar J. 2017;16:130. PubMed http://dx.doi.org/10.1186/s12936-017-1783-2

57. Laufer MK, Plowe CV. Withdrawing antimalarial drugs: impact on parasite resistance and implications for malaria treatment policies. Drug Resist Updat. 2004;7:279–88. PubMed http://dx.doi.org/10.1016/j.drup.2004.08.003

58. Lobo E, de Sousa B, Rosa S, Figueiredo P, Lobo L, Pateira S, et al. Prevalence of pfmdr1 alleles associated with artemether-lumefantrine tolerance/resistance in Maputo before and after the implementation of artemisinin-based combination therapy. Malar J. 2014;13:300. PubMed http://dx.doi.org/10.1186/1475-2875-13-300

59. Desai M, Gutman J, Taylor SM, Wiegand RE, Khairallah C, Kayentao K, et al. Impact of Sulfadoxine-Pyrimethamine Resistance on Effectiveness of Intermittent Preventive Therapy for Malaria in Pregnancy at Clearing Infections and Preventing Low Birth Weight. Clin Infect Dis. 2016;62:323–33. PubMed http://dx.doi.org/10.1093/cid/civ881

60. Gutman J, Mwandama D, Wiegand RE, Ali D, Mathanga DP, Skarbinski J. Effectiveness of intermittent preventive treatment with sulfadoxine-pyrimethamine during pregnancy on maternal and birth outcomes in Machinga district, Malawi. J Infect Dis. 2013;208:907–16. PubMed http://dx.doi.org/10.1093/infdis/jit276

61. Kayentao K, Garner P, van Eijk AM, Naidoo I, Roper C, Mulokozi A, et al. Intermittent preventive therapy for malaria during pregnancy using 2 vs 3 or more doses of sulfadoxine-pyrimethamine and risk of low birth weight in Africa: systematic review and meta-analysis. JAMA. 2013;309:594–604. PubMed http://dx.doi.org/10.1001/jama.2012.216231

62. World Health Organization. Intermittent preventive treatment of malaria in pregnancy using sulfadoxine-pyrimethamine (IPTp-sulfadoxine-pyrimethamine). Updated WHO Policy Recommendation. 2012. Geneva: The Organization. p. 1–3.

63. Laufer MK, van Oosterhout JJ, Thesing PC, Thumba F, Zijlstra EE, Graham SM, et al. Impact of HIV-associated immunosuppression on malaria infection and disease in Malawi. J Infect Dis. 2006;193:872–8. PubMed http://dx.doi.org/10.1086/500245

64. Artimovich E, Schneider K, Taylor TE, Kublin JG, Dzinjalamala FK, Escalante AA, et al. Persistence of Sulfadoxine-Pyrimethamine Resistance Despite Reduction of Drug Pressure in Malawi. J Infect Dis. 2015;212:694–701. PubMed http://dx.doi.org/10.1093/infdis/jiv078
